# Role of the bed nucleus of the stria terminalis in feeding

**DOI:** 10.3389/fnut.2025.1598703

**Published:** 2025-09-05

**Authors:** Keyan Wang, Wenbo Guo, Jiaxiang Xu, Yingjie Kuang, Xiqi Chen

**Affiliations:** ^1^College of Health Sciences, Shandong University of Traditional Chinese Medicine, Jinan, China; ^2^Department of Emergency Surgery, Affiliated Hospital of Shandong University of Traditional Chinese Medicine, Jinan, China; ^3^First School of Clinical Medicine, Shandong University of Traditional Chinese Medicine, Jinan, China

**Keywords:** BNST, eating behavior, neuropeptide, neural circuit, appetite regulation

## Abstract

As an important part of the limbic system of the forebrain, the bed nucleus of the stria terminalis (BNST) plays a key role in stress response, emotion regulation, and motivational behavior. Recent studies have demonstrated that BNST plays a crucial role in the pathological process of appetite regulation, food selection, and eating disorders by integrating metabolic signals, reward feedback, and stress input. This article systematically reviews the anatomical subdivision, neural loop characteristics, and multimodal mechanisms of the BNST in food intake regulation; discusses its association with obesity, anorexia nervosa, and other diseases; and explores potential therapeutic strategies targeting the BNST.

## 1 Introduction

Feeding regulation involves the integrated processing of homeostatic needs, reward-related mechanisms, and environmental challenges. The bed nucleus of the stria terminalis (BNST) is a basal forebrain structure located posterior to the nucleus accumbens, anterior to the thalamus, medial to the dorsal striatum, and dorsal to the ventral pallidum and the dorsomedial preoptic area. This forebrain structure comprises 12–20 distinct subdomains containing heterogeneous neuronal populations classified by (1) morphological characteristics, (2) neurotransmitter/neuropeptide profiles, (3) biophysical properties, and (4) efferent/afferent connectivity patterns. The anatomical complexity of the BNST closely matches the many behavioral, autonomic and endocrine functions it supports, among which frequently discussed are anxiety (1, 2), stress (3, 4), aversion and memory (5, 6), addiction (7, 8), social behavior (9), appetite control (10), cardiovascular modulation (11), and hormone release (12). Understanding how the BNST achieves such a diverse range of functions, and the molecular and loop mechanisms behind them, remains a major challenge.

Advanced precision neuroscience methodologies available at the time of study enable highly specific exploration of neural dynamics at the cellular level. By integrating optogenetic and chemogenetic manipulations with multiphoton imaging, fiber photometry, and viral vector-mediated genetic targeting, researchers can achieve precise analyses of genetically defined neuronal populations and circuit-level interactions. This technological synergy has significantly advanced the dissection of the BNST microarchitecture and its functional coding mechanisms. In this study, we aim to review current insights into BNST subdomain specialization, connectivity patterns, and molecular determinants, offering a comprehensive perspective on its neuromodulatory role in homeostatic feeding regulation.

## 2 Structure of the BNST

**Figure 1:**
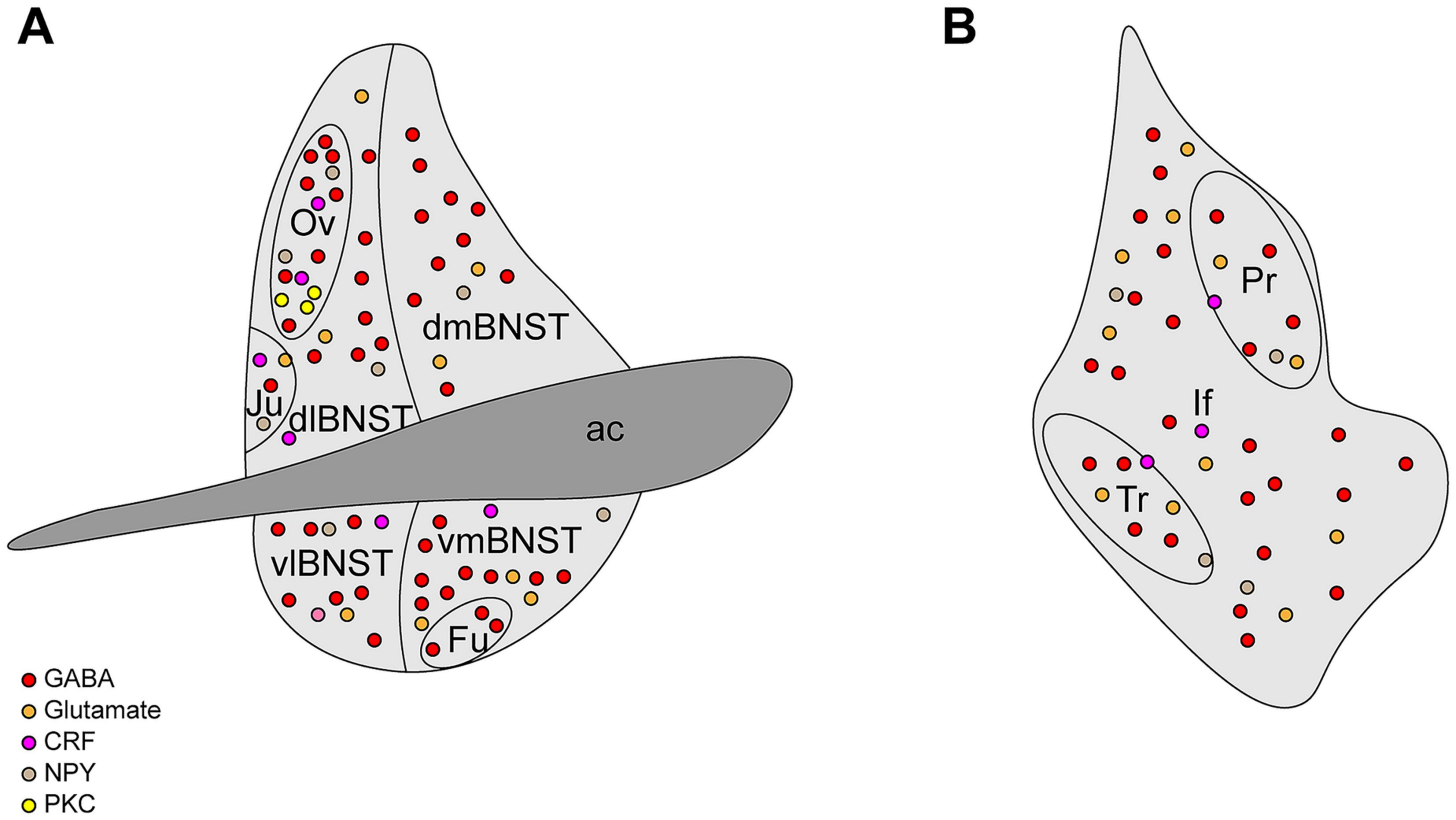
Structural diagram of the BNST. **(A)** The anterior BNST can be divided into dorsolateral BNST (dlBNST), dorsomedial BNST (dmBNST), ventrolateral (vlBNST), ventromedial (vmBNST), oval nucleus (Ov), juxtacapsular nucleus (Ju), and fusiform nucleus (Fu). **(B)** The posterior BNST can be divided into the principal nucleus (Pr), interfascicular nucleus (If), and transverse nucleus (Tr).

The BNST is a compact yet highly heterogeneous limbic structure in the ventral forebrain, playing a crucial role in integrating and coordinating stress-related behaviors. Composed of multiple subnuclei and neurochemically distinct cell populations, it forms part of the “extended amygdala” alongside the central amygdala (CeA) and the caudal nucleus accumbens (NAc) shell. BNST connectivity extends to other limbic regions, including the amygdala complex, the hypothalamic nuclei, the hippocampus, and the midbrain structures, contributing to emotion regulation, emotional learning, and stress responses. Acting as a key integrative hub, the BNST processes sensory inputs and transmits signals to central neuroendocrine and autonomic centers, ensuring appropriate physiological and behavioral adaptations. In rodents, the BNST is positioned anterior to the anterior fontanel, bordered by the anterior commissure, the lateral ventricles, and the internal capsule-associated regions, extending from the nucleus accumbens and lateral septal nucleus to the anterior hypothalamus. Despite its small size (~0.4 mm^3^ in mice), the BNST is subdivided into 12–20 nuclei, encompassing over 15 distinct cell types (13, 14). The BNST demonstrates distinct anatomical organization along its anterior–posterior axis, traditionally divided into the anterior and posterior regions (15). The anterior BNST can be further divided into the dorsal (dBNST) and ventral (vBNST) regions, which maintain ventrolateral continuity and are further subdivided into the lateral and medial portions. The dBNST includes the dorsolateral (dlBNST) and dorsomedial (dmBNST) regions, defined by their position relative to the stria terminalis, while the vBNST comprises ventrolateral (vlBNST) and ventromedial (vmBNST) subdivisions. Notable nuclei within dlBNST include the oval nucleus and juxtacapsular nucleus, whereas the vBNST contains the fusiform nucleus (Figure 1A). The posterior BNST comprises three well-defined nuclei (principal, interfascicular, and transverse) with distinct neurochemical profiles (Figure 1B), characterized by particularly high expression of sex hormone receptors, which are critical for regulating reproductive behaviors and defensive responses (4, 16).

## 3 BNST afferent inputs and feeding behavior regulation

**Figure 2:**
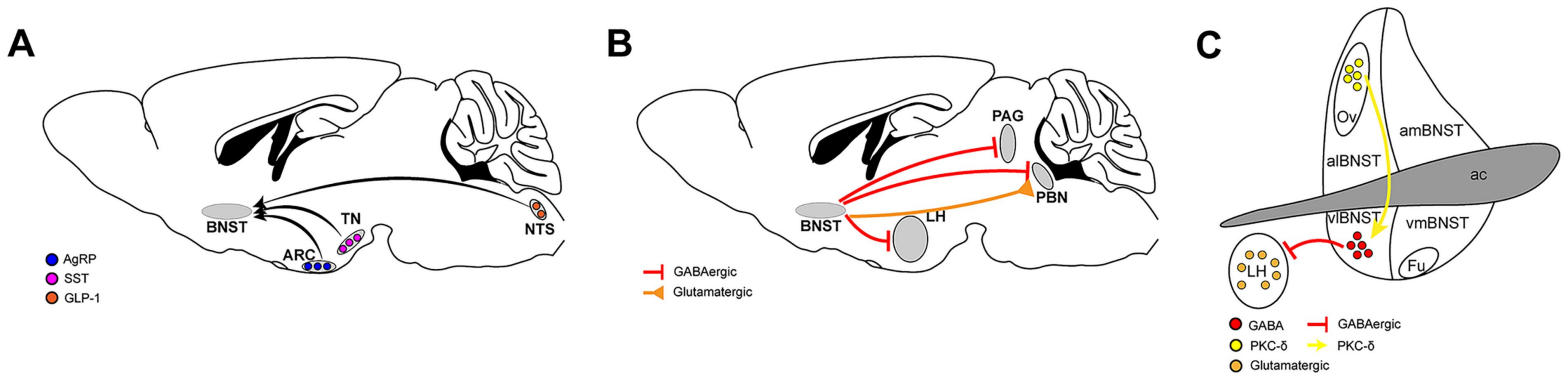
Neural circuits involving the BNST in feeding regulation. **(A)** The upstream brain regions of the BNST that regulate food intake, nucleus tractus solitarius (NTS), arcuate nucleus (ARC), tuberous nucleus (TN), Agouti-related peptide (AgRP), Glucagon-like peptide-1 (GLP-1), Somatostatin (SST). **(B)** The BNST transmits signals to downstream brain regions to regulate food intake, parabrachial nucleus (PBN), lateral hypothalamus (LH), periaqueductal gray (PAG). **(C)** The microcircuits within the BNST that regulate food intake, protein kinase C delta (PKC-*δ*).

The BNST receives input signals from multiple upstream brain regions to regulate eating (Figure 2A).

The BNST brain areas express a variety of neuropeptides and multiple neuropeptide receptors, responding to neurotransmitters transmitted from multiple brain areas. Glucagon-like peptide-1 (GLP-1) injected into the brain reduces food intake. Similarly, activating preproglucagon (PPG) cells in the brain, which synthesize GLP-1, reduces food intake. PPG neurons that express GLP-1 in the nucleus tractus solitarius (NTS) are widely believed to play an important role in regulating food intake, weight, and stress responses (17, 18). These neurons project to areas across the brain where many GLP-1 receptors (GLP-1R) are expressed. Both the dorsal and ventral parts of the BNST receive projections from PPG neurons in the NTS. Direct injection of exogenous GLP-1 into the BNST effectively reduces food intake in mice. Blocking the GLP-1 receptor in BNST by microinjection of the GLP-1R antagonist exendin (9–39) significantly increases acute food intake, thus confirming that BNST GLP-1R is a physiologically relevant target of endogenous GLP-1 (19).

Agouti-related peptide (AgRP), a hypothalamic neuropeptide synthesized in ventromedial arcuate nucleus (ARC) neurons co-expressing neuropeptide Y (NPY), orchestrates energy balance through dual orexigenic actions comprising appetite potentiation coupled with metabolic rate suppression. This evolutionarily conserved mediator, exclusively produced by AgRP/NPY co-localized neuronal populations, exhibits the most robust and sustained hunger-promoting effects among central feeding regulators. Anatomically, AgRP neuronal projections to the anterior BNST (aBNST) constitute a critical circuit mediating feeding potentiation, with optogenetic activation of aBNST-targeting AgRP neurons triggering hyperphagia in murine models. Crucially, AgRP’s neuromodulatory function demonstrates obligatory NPY dependence, as evidenced by complete ablation of its feeding-modulatory capacity in NPY-knockout rodents (20).

Somatostatin (SST) neurons in the tuberous nucleus (TN) play a crucial role in regulating food intake in mice. These hunger-activated neurons project to the BNST and release gamma-aminobutyric acid (GABA), which promotes food intake in mice by inhibiting the cellular activity of BNST. Conversely, inhibition of this group of SST neurons reduces food intake (21).

The BNST’s reception of signals from various upstream sources exerts diverse impacts on the feeding behavior of mice. This finding might be associated with differences in the subregions and cell types that receive these signals.

## 4 BNST downstream projections and feeding behavior regulation

The BNST not only receives neuronal projections from multiple brain areas but also projects neurons to many brain areas in order to regulate eating, especially the mid-posterior part of the brain (Figure 2B).

In mammals, the parabrachial nucleus (PBN), a pontine structure integrating visceral and sensory inputs, regulates food intake and threat assessment by encoding metabolic needs. Studies have shown that there are two different groups of neurons in the BNST that project to PBN, and both have an impact on eating behavior. One group consists of GABAergic neurons. Activation of the neuron terminals projecting from the BNST to the PBN brain area will increase the feeding of satiated mice in a short period of time, while inhibition of this loop will cause reduced feeding of hungry mice. The other nucleus is composed of glutamatergic neurons. Contrary to the effect of GABAergic nuclei, activating their nerve terminals in PBN will inhibit the feeding behavior of mice in a short period of time, while chemical inhibition of this loop will increase the food intake of satiated mice (22).

The lateral hypothalamus (LH) is an important neural substrate for motivated behaviors, including eating. A large number of vesicular GABA transporter (VGAT) neurons in the BNST project to the LH, mainly targeting glutamatergic neurons. When activated, these VGAT neuron terminals induce greedy feeding behavior in satiated mice, while inhibition significantly reduces food intake in hungry mice. However, it is worth noting that suppression of this loop significantly increased aversion to the light pairing chamber in the position-preference experiment, so it is difficult to say whether the mice’s reduced eating was simply due to a change in food preference or because of an aversion to the entire environment (23).

The periaqueductal gray (PAG) constitutes a pivotal integration node mediating adaptive survival responses to environmental challenges through its quadripartite columnar organization (24). Within this framework, the ventrolateral PAG (vlPAG) emerges as a specialized mediator of defensive states, particularly governing threat-evoked freezing responses in mammals, which is an evolutionarily conserved mechanism balancing predation risk during foraging activities (25). Moreover, vlPAG directly receives projections from multiple brain regions involved in feeding regulation. The BNST sends monosynaptic GABAergic inputs to GABAergic cells in the vlPAG. Photogenetic activation of the terminals of these GABAergic neurons can induce feeding behavior. Bilateral inhibition of this loop does not affect the feeding behavior of satiated mice but significantly reduces the food intake of mice fasted for 24 h (26).

It can be observed that the BNST mainly promotes feeding in mice by releasing GABAergic signals to downstream brain regions.

## 5 Microcircuits regulating eating in the BNST

Because the BNST is composed of many subregions, there are also neuronal projections between them, one of which regulates eating. The oval region of the BNST (ovBNST) is a functionally diverse brain area involved in regulating multiple emotional and physiological reactions. Its role in social behavior, emotional cognition, and stress response is a topic of great interest in current neuroscience research. Current studies have shown that the ovBNST also plays an important role in regulating eating behavior. Inflammation-induced anorexia activates protein kinase C delta (PKC-*δ*) neurons in the ovBNST brain area. Photogenetic activation of these neurons results in a significant decrease in the amount of food consumed by mice during the stimulation period, whereas chemical genetic inhibition of these neurons can increase the total amount of food consumed by mice. These neurons mainly project to GABAergic neurons in vlBNST that regulate eating, and these GABAergic neurons primarily project to the LH (Figure 2C). In other words, the ovBNST regulates eating through the regulation of the vlBNST-LH loop (27).

## 6 BNST abnormalities and eating disorders

Corticotropin-releasing factor (CRF) is a neuropeptide that regulates endocrine and behavioral stress responses. Overexpression or knock-out of CRF in the mouse brain affects feeding behavior of mice. The BNST contains abundant CRF receptor subtypes, namely corticotropin-releasing factor receptor type 1 (CRFR1) and corticotropin-releasing factor receptor type 2 (CRFR2). Studies have shown that CRFR1 receptors in the BNST are involved in regulating overeating behavior due to stress, while CRFR2 receptors regulate appetite loss.

In rat experiments, researchers found that rats exposed to stress exhibited increased food intake, which was reduced by the administration of specific CRFR1 receptor antagonists. This finding suggests that activation of CRFR1 receptors in BNST leads to increased eating. This activation is often accompanied by individuals being exposed to some environmental stress, which suggests that an abnormal increase in activation of CRFR1 receptors in BNST may lead to individuals suffering from binge eating and obesity (28). In other experiments, researchers found that rats developed anorexia after experiencing acute restraint, and injecting CRFR2 receptor antagonists into the BNST eliminated the reduced eating after restraint in rats, suggesting that CRFR2 in the BNST plays an important role in stress-induced anorexia (29).

These studies will help to provide a more comprehensive understanding of the role of BNST in eating behavior and provide new ideas and targets for the treatment of eating disorders.

## 7 Discussion

The BNST serves as a “decision-making center” for regulating eating behavior by integrating metabolic, reward, and stress signals. Its dysfunction is closely related to a variety of eating disorders, and intervention strategies targeting specific BNST subunits or neurotransmitter systems have broad prospects. Future research should combine cross-species models and clinical transformation to promote the practical application of BNST-related therapies.
